# Rural Easy Japanese as a Method of Mitigating Language Barriers Among Foreigners Seeking Medical Care in Rural Japan

**DOI:** 10.7759/cureus.26693

**Published:** 2022-07-09

**Authors:** Ryuichi Ohta, Chiaki Sano

**Affiliations:** 1 Community Care, Unnan City Hospital, Shimane, JPN; 2 Community Medicine Management, Shimane University Faculty of Medicine, Izumo, JPN

**Keywords:** medical education, japan, foreigner, social isolation, rural, language barriers, easy japanese, covid-19

## Abstract

Mitigating difficulties in communication is vital in multicultural societies. Particularly, foreigners face greater communication difficulties because of language barriers, posing a challenge to both themselves and indigenous people. In Japan, the increase in the number of foreigners has driven the use of Easy Japanese, a free online program of Japanese language lessons. Easy Japanese can facilitate communication between medical professionals and foreigners. Easy Japanese is mainly characterized by short sentences, an upfront conclusion, and avoiding polite Japanese words when collaborating with translators. This communication method should prevail not only in urban areas but also in rural areas, and can help individuals in rural contexts prepare for an increase in the number of foreigners.

## Introduction

Mitigating difficulties in communication is vital in multicultural societies. Particularly, foreigners face greater communication difficulties because of language barriers, posing a challenge to them [1]. Misunderstandings can cause conflicts in communities, impinging on community members’ daily lives and health conditions [1]. Hence, communication difficulties should be mitigated through translation systems and a better understanding of multicultural differences within communities [2].

Foreigners face additional issues in communication in rural Japan, which are related to the rural culture. With an increasing number of foreigners working in rural areas, their communication difficulties with indigenous people may be caused by both language barriers and issues related to Japanese rural culture, such as the monogamous culture [3,4]. Unlike urban areas, rural Japan may not be used to multicultural situations. Monogamy restricted to one’s own community could be a norm in rural communities, leading to the exclusion of foreigners [5]. Thus, foreigners may not be easily accepted under rural Japanese culture.

## Technical report

Easy Japanese

**Figure 1:**
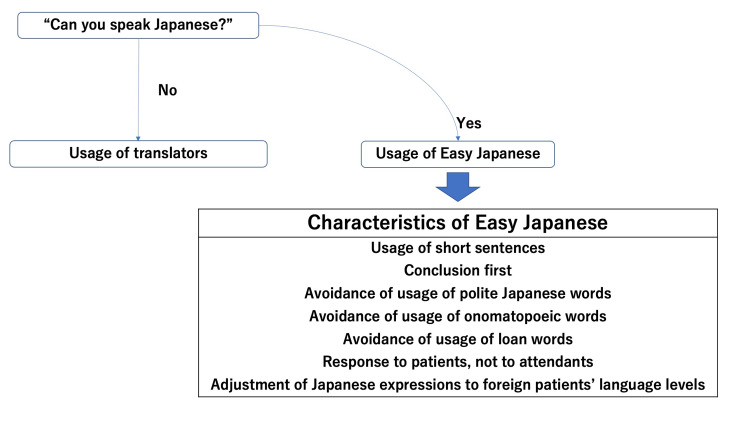
Basic rules of Easy Japanese in medical fields

In Japan, an increase in the number of foreigners has driven the use of Easy Japanese among them. Easy Japanese was established in Japan to mitigate foreigners’ difficulty in understanding the Japanese language [6]. Easy Japanese is mainly characterized by short sentences, the conclusion at first, and avoiding polite Japanese words when collaborating with translators (Figure 1).

The use of medical interpreters and facilitating methods such as Easy Japanese became widespread, particularly in urban areas, during the Tokyo Olympics 2020 [7].

Easy Japanese can be used in medical fields, particularly to mitigate the fears and anxieties of foreigners. Foreigners may experience anxiety and fear when visiting rural primary care professionals and family physicians if they cannot speak the country’s language [8]. Miscommunication between foreign patients and rural primary care professionals and family physicians could cause various kinds of damage to patients’ health conditions, such as misdiagnosis and inappropriate treatment [9,10]. To appropriately address foreign patients’ health conditions, rural primary care professionals and family physicians should use effective communication methods with them, such as Easy Japanese.

In medical consultations, the use of Easy Japanese could facilitate better care of foreigners in medical institutions by soothing any anxiety that may occur between foreigners and Japanese rural primary care professionals and family physicians. Both parties may feel anxious about communicating medical complaints. By using the rules of Easy Japanese, both parties can communicate smoothly based on their own skills and by collaborating with translators (Figure 1).

For example, rural primary care professionals and family physicians first assess the perceptions of foreign patients about their Japanese language abilities. If a foreign patient does not have a good command of the Japanese language, rural primary care professionals and family physicians can use translators to avoid the risk of miscommunication. However, due to a lack of medical translators, in situations when foreign patients have a basic knowledge of Japanese, Easy Japanese can be used for quick communication between the two parties [11].

There are several rules when using Easy Japanese (Figure 1). Japanese people tend to speak in long sentences, which can be difficult for foreigners to understand. Therefore, sentences should be short and state the conclusion first. In addition, the usage of polite, onomatopoeic, and Japanese loan words should be avoided as these words are specific to Japanese culture and can make it even more challenging for foreigners to understand.

However, rural primary care professionals and family physicians should not forget to directly communicate with foreign patients; instead of focusing on patients’ attendants who can speak Japanese, they should respect patients’ ability to converse in Japanese because some foreigners speak Japanese fluently.

## Discussion

In both urban and rural areas, there is an increasing need to adopt Easy Japanese for effective communication with foreigners, particularly in the medical field. Foreigners in rural communities stated that they are not used to communicating while wearing masks, a pre-requisite following the outbreak of COVID-19, as facial expressions form an important aspect of understanding conversations [12]. In the medical field, where patients are required to explain their symptoms, communication may fail if medical professionals are not conversant with communication methods such as Easy Japanese [13]. This may contribute to the patient's anxiety regarding their symptoms and treatment plans.

To ensure effective assimilation of foreigners from different countries, it is necessary to ensure adequate language skills, alleviate fear and anxiety, secure rural primary care, and involve family physicians and other medical professionals when providing medical care to foreigners in Japan. At present, medical professionals’ understanding of Easy Japanese and the health conditions of foreigners may not be enough to ease foreigners’ difficulties, particularly in rural Japan [6]. Medical professionals must prepare to deliver medical care in multicultural societies that accept foreigners and enrich the Japanese culture, particularly in rural contexts [14]. Owing to the lack of medical resources in rural Japan, foreigners feel more anxious regarding medical treatment in Japan [15]. The presence of rural medical professionals who can overcome language barriers is essential to rural medicine [16].

Rural primary care professionals and family physicians should become familiar with the language and communication barriers that foreigners experience in rural contexts. In addition, they should share these problems in interprofessional collaboration to improve the future of rural medicine and the rural community [17,18]. Each rural community has a different background regarding issues with foreigners and health-seeking behaviors [19,20]. Hence, the methods in Easy Japanese should be further reviewed based on the challenges faced by foreigners and indigenous people in rural contexts, with rural primary care professionals and family physicians playing a leading role in facilitating communication.

## Conclusions

This report highlights the importance and need for effective communication with foreigners through the usage of facilitating tools such as Easy Japanese. As Japan is becoming increasingly multicultural, medical professionals should consider adapting the way they communicate with foreign patients to ease their anxieties and fears to preserve patients’ health and provide the most appropriate treatment. Easy Japanese could be used to promote effective communication between foreign patients and medical professionals.
